# Microfluidic PCR and network analysis reveals complex tick-borne pathogen interactions in the tropics

**DOI:** 10.1186/s13071-023-06098-0

**Published:** 2024-01-04

**Authors:** Cristian Díaz-Corona, Lisset Roblejo-Arias, Elianne Piloto-Sardiñas, Adrian A. Díaz-Sánchez, Angélique Foucault-Simonin, Clemence Galon, Alejandra Wu-Chuang, Lourdes Mateos-Hernández, Zbigniew Zając, Joanna Kulisz, Aneta Wozniak, María Karla Castro-Montes de Oca, Evelyn Lobo-Rivero, Dasiel Obregón, Sara Moutailler, Belkis Corona-González, Alejandro Cabezas-Cruz

**Affiliations:** 1 Direction of Animal Health, National Center for Animal and Plant Health, Carretera de Tapaste y Autopista Nacional, Apartado Postal 10, 32700 San José de Las Lajas, Mayabeque Cuba; 2 UMR BIPAR, Laboratoire de Santé Animale, ANSES, INRAE, Ecole Nationale Vétérinaire d’Alfort, 94700 Maisons-Alfort, France; 3 Department of Biology, University of Saskatchewan, 112 Science Place, Saskatoon, SK S7N 5E2 Canada; 4 Department of Biology and Parasitology, Medical University of Lublin, Radziwiłłowska 11 St, 20-080 Lublin, Poland; 5 School of Environmental Sciences, University of Guelph, Guelph, ON N1G 2W1 Canada

## Abstract

**Background:**

Ixodid ticks, particularly *Rhipicephalus sanguineus* s.l., are important vectors of various disease-causing agents in dogs and humans in Cuba. However, our understading of interactions among tick-borne pathogens (TBPs) in infected dogs or the vector *R. sanguineus* s.l. remains limited. This study integrates microfluidic-based high-throughput real-time PCR data, Yule's Q statistic, and network analysis to elucidate pathogen-pathogen interactions in dogs and ticks in tropical western Cuba.

**Methods:**

A cross-sectional study involving 46 client-owned dogs was conducted. Blood samples were collected from these dogs, and ticks infesting the same dogs were morphologically and molecularly identified. Nucleic acids were extracted from both canine blood and tick samples. Microfluidic-based high-throughput real-time PCR was employed to detect 25 bacterial species, 10 parasite species, 6 bacterial genera, and 4 parasite taxa, as well as to confirm the identity of the collected ticks. Validation was performed through end-point PCR assays and DNA sequencing analysis. Yule's Q statistic and network analysis were used to analyse the associations between different TBP species based on binary presence-absence data.

**Results:**

The study revealed a high prevalence of TBPs in both dogs and *R. sanguineus* s.l., the only tick species found on the dogs. *Hepatozoon canis* and *Ehrlichia canis* were among the most common pathogens detected. Co-infections were observed, notably between *E. canis* and *H. canis*. Significant correlations were found between the presence of *Anaplasma platys* and *H. canis* in both dogs and ticks. A complex co-occurrence network among haemoparasite species was identified, highlighting potential facilitative and inhibitory roles. Notably, *H. canis* was found as a highly interconnected node, exhibiting significant positive associations with various taxa, including *A. platys*, and *E. canis*, suggesting facilitative interactions among these pathogens. Phylogenetic analysis showed genetic diversity in the detected TBPs.

**Conclusions:**

Overall, this research enhances our understanding of TBPs in Cuba, providing insights into their prevalence, associations, and genetic diversity, with implications for disease surveillance and management.

**Graphical abstract:**

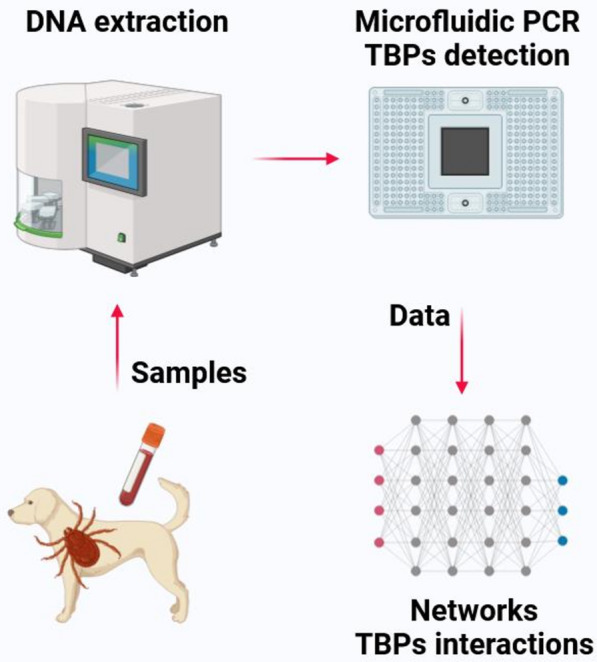

**Supplementary Information:**

The online version contains supplementary material available at 10.1186/s13071-023-06098-0.

## Background

Tick-borne diseases (TBDs) constitute a serious health risk for both humans and animals on a global scale. In recent decades, the increasing prevalence and transmission of tick-borne pathogens (TBPs) have become major concerns for public health and veterinary medicine [1]. Notably, ixodid ticks can transmit a wider array of disease-causing pathogens (e.g. bacteria, viruses, and protozoa) compared to any other arthropod vector [2]. In addition, ticks can harbour multiple pathogens simultaneously, leading to coinfections, which can make diagnosis and treatment challenging [3].

Dogs serve as valuable sentinels for the monitoring of prevalence, diversity, and distribution of TBPs, particularly tick-borne protozoan and bacterial agents, through scientific research and surveillance efforts [4]. Particularly, domestic dogs play a valuable role as animal sentinels due to their special bond with humans and shared household and recreational risk factors [5]. The brown dog tick, *Rhipicephalus sanguineus* sensu lato (s.l.) (Latreille, 1806), also known as the kennel tick or pan-tropical dog tick, is likely the most widespread ixodid tick species globally [6]. The primary hosts of this tick species are mainly canids, and it is a well-known competent vector of several pathogens causing diseases in dogs, including babesiosis, ehrlichiosis, and hepatozoonosis, as well as rickettsiosis, which can affect both dogs and humans [7].

In Cuba, the distribution of *R. sanguineus* tick species extends primarily to the western region, encompassing provinces such as La Habana, Mayabeque, and Pinar del Río [8]. Previous research work has provided some information about the occurrence of TBPs in dogs from Cuba, including *Anaplasma platys*, *Ehrlichia canis*, *Hepatozoon canis*, *Rickettsia felis*, *Mycoplasma haemocanis*, and *Candidatus* *Mycoplasma haematoparvum* [9–12]. Additionally, the presence of *Babesia vogeli* and *Rickettsia amblyommatis* has been observed in *R. sanguineus* s.l. and *Amblyomma mixtum* ticks collected from dogs, respectively [13, 14].

The microfluidic-based high-throughput real-time PCR systems have emerged as advanced molecular diagnostic tools, reshaping the detection and quantification of genetic material such as DNA or RNA in biological samples [15]. These systems offer a unique ability to simultaneously detect a diverse array of TBPs with high sensitivity and specificity using minimal DNA volumes, representing an innovative alternative to large-scale epidemiological surveys of TBPs compared to standard PCR assays [16, 17]. The integration of microfluidic-based high-throughput real-time PCR data with statistical methods that measure the association between two or more binary or nominal variables presents a powerful approach to enhance our understanding of TBPs and their complex interactions. Particularly, Yule’s Q statistic [18], designed to assess the strength and direction of associations in binary data, aligns seamlessly with the output of high-throughput PCR systems.

This study aims to illuminate the diverse landscape of TBPs present in domestic dogs and ticks across the western region of Cuba through the utilization of microfluidic-based high-throughput real-time PCR detection. In addition, this study endeavours to decipher the intricate associations among different pathogen pairs through the integration of cutting-edge molecular diagnostic tools and innovative statistical approaches, contributing to the formulation of effective strategies for disease surveillance, control, and management.

## Methods

### Study design and sample collection

#### Blood sample collection

A cross-sectional study was conducted in different localities of provinces La Habana and Mayabeque in Cuba between January 2022 and August 2022. The study involved the collection of blood samples from 46 client-owned dogs (32 male and 14 females). The dogs were randomly selected, representing different age and breeds categories, with a predominant presence of middle-aged and crossbred dogs. During the sample collection process, a veterinarian performed physical examination on the dogs. This examination included recording their body temperature, hydration status, and presence of skin lesions. The blood samples were drawn aseptically from the jugular vein using sterile Vacutainer needles and EDTA tubes (Becton-Dickinson Vacutainer Systems in Franklin Lakes, NJ, USA). The collected blood samples were then stored at 4 °C and within 24 h from collection DNA extraction was performed (see below).

#### Tick collection and taxonomic identification

The sampled dogs were inspected manually for tick infestation, all ticks attached to the skin were removed, and a representative sample of up to ten ticks per animal was collected for further analysis. The collected ticks were placed in labelled tubes and transported alive to the laboratory for identification to development stage and to species level. The ticks were identified morphologically under a dissecting stereoscopic microscope (Carl Zeiss Light Microscopy, Göttingen, Germany) according to the standard taxonomic keys described by Walker and Keirans [19]. Although the collected specimens included immature ticks, only the adults were identified to the species level. Once identified, specimens of each tick group were preserved in 70% ethanol using 1.5-ml plastic sterile tubes and stored at—80 °C until DNA extraction. An adult female tick belonging to the species *R. sanguineus* s.l. was selected for further analysis from each sampled animal.

#### Nucleic acids extraction from canine blood and tick samples

Total nucleic acid was extracted from each EDTA-anticoagulated blood sample (300 μl) using the Wizard^®^ Genomic DNA Purification kit (Promega, Madison, WI, USA) following the manufacturer’s instructions. Each tick sample was rinsed three times in double-distilled water to eliminate residual 70% alcohol, dried on sterile filter paper, and sectioned using a sterile scalpel in Petri dishes. Subsequently, the excised tick fragments were transferred to a MagNA Lyser tube, along with ceramic beads and 100 μl of phosphate-buffered saline (PBS) (Sigma, St. Louis, MO, USA). The homogenization process was carried out using a Roche MagNA Lyser (Roche Molecular Diagnostics) at a speed of 5000 rpm (5 × 60 s). The resultant homogenized aliquots of tick tissue were transferred to sterile tubes for subsequent DNA extraction. Afterwards, tick homogenates were subjected to total nucleic acid extraction using the Wizard^®^ Genomic DNA Purification kit (Promega, Madison, WI, USA), following the manufacturer’s instructions. To ensure the integrity of the procedure and minimize the risk of cross-contamination, negative controls were prepared concurrently by adding 300 μl PBS (Sigma, St. Louis, MO, USA) to each batch of 20 samples. The quantitative and qualitative evaluation of the extracted total nucleic acid was determined using a Colibri Microvolume Spectrophotometer (Titertek-Berthold, Pforzheim, Germany). The extracted nucleic acid samples were stored at − 20 °C until further use.

### Molecular detection of tick-borne pathogens

#### DNA pre-amplification for microfluidic real-time PCR

To enhance pathogen DNA detection, total DNA was initially pre-amplified using the PreAmp Master Mix (Standard Biotools, San Francisco, CA, USA), following the manufacturer's guidelines. Primers (Additional file 3: Table S1), excluding those targeting tick DNA and controls, were combined in equal volumes to create a pooled primer mix with a final concentration of 200 nM. The reaction was carried out in a 5-μl volume, consisting of 1 μl Perfecta Preamp 5 ×, 1.25 μl pooled primer mix, 1.5 μl distilled water, and 1.25 μl DNA. The thermocycling programme consisted of one cycle at 95 °C for 2 min, followed by 14 cycles at 95 °C for 15 s and 60 °C for 4 min. After the cycling programme, the reactions were diluted 1:10 in Milli-Q ultrapure water. All pre-amplified DNA samples were stored at − 20 °C until further use.

#### Microfluidic real-time PCR assay

The study aimed to assess the presence of various bacteria, parasites, and ticks within a sample using high-throughput microfluidic real-time PCR amplification. The following organisms were investigated:

- Bacterial species (*n* = 25): *Mycoplasma haemofelis*, *M. ovis*, *M. haemocanis*, *Candidatus* *Mycoplasma haematoparvum*, *Borrelia miyamotoi*, *Anaplasma marginale*, *A. platys*, *A. phagocytophilum*, *A. bovis*, *Ehrlichia ewingii*, *E. chaffeensis*, *E. canis*, *Neoehrlichia mikurensis*, *Rickettsia conorii*, *R. slovaca*, *R. massiliae*, *R. helvetica*, *R. aeschlimannii*, *R. felis, R. rickettsii*, *Bartonella henselae*, *Francisella tularensis*, *Francisella*-like endosymbionts, *Coxiella*-like endosymbionts, and *C. burnetii*.
- Parasite species (*n* = 10): *Babesia microti*, *B. canis* (3 subspecies), *B. ovis*, *B. divergens*, *Babesia* sp. EU1, *Hepatozoon canis*, *H. americanum*, *Cytauxzoon felis*, *Rangelia vitalii*, and *Leishmania infantum*.
- Bacterial genera (*n* = 6): *Bartonella*, *Borrelia*, *Anaplasma*, *Ehrlichia*, *Rickettsia*, and *Mycoplasma*.
- Parasite taxa (*n* = 4): *Apicomplexa*, *Theileria*, *Hepatozoon*, and *Leishmania*.
- Tick species (*n* = 1): *Rhipicephalus sanguineus* s.l.

The assessment was conducted using 48.48 Dynamic Array™ IFC chips (Standard Biotools, CA, United States) in the BioMark™ real-time PCR system (Standard Biotools, San Francisco, CA, USA). Each chip allowed the allocation of 48 PCR mixtures and 48 samples into individual wells. The real-time PCR reactions were performed in individual chambers using on-chip microfluidics assembly. The thermal cycling process generated a total of 2304 individual reactions. The amplification process utilized 6-carboxyfluorescein (FAM)- and black hole quencher (BHQ1)-labelled TaqMan probes with TaqMan Gene expression master mix, following the manufacturer's instructions (Applied Biosystems, Courtaboeuf, France). The thermocycling programme consisted of an initial step of 2 min at 50 °C, followed by 10 min at 95 °C and 40 cycles of two-step amplification: 15 s at 95 °C and 1 min at 60 °C. A negative water control was included for each chip. To confirm the absence of PCR inhibitors in the tested samples, *Escherichia coli* strain EDL933 DNA was added to each sample as an internal inhibition control, using specific primers and a probe for the *E. coli eae* gene. In addition, the amplification of *R. sanguineus* s.l. DNA among tick samples validated the presence of the tested tick species. Additional file 3: Table S1 contains information about the target genes and primer sequences used for amplification. The development of this new high-performance tool, based on real-time microfluidic PCR, involved several crucial elements: sensitivity testing, specificity evaluation, and implementation of essential controls. Grech-Angelini et al. [19] and Michelet et al. [14] have provided detailed descriptions of these aspects in their research.

### Validation of microfluidic real-time PCR system results

#### Endpoint PCR assays

To validate the microfluidic real-time PCR results, a subset of positive samples infected with specific TBPs underwent additional conventional and nested PCR assays using different species-specific primers from those used in the BioMark™ system. The cycling conditions and primers for sequence analysis are listed in Additional file 4: Table S2.

#### DNA sequencing analysis

The obtained amplicons were submitted for sequencing at Eurofins MWG Operon (Ebersberg, Germany). The resulting sequences were assembled using the BioEdit software (Ibis Biosciences, Carlsbad, CA, USA). Subsequently, these sequences were analysed to identify the targeted TBPs by conducting a search against the GenBank database using the Basic Local Alignment Sequence Tool (BLASTn) search engine (http://blast.ncbi.nlm.nih.gov/Blast.cgi) [20] provided by the National Center for Biotechnology Information (NCBI, Bethesda, MD, USA). Theoretical translation of nucleotide sequences into amino acid sequences using the ExPASy translate tool, available on the ExPASy molecular biology server (http://www.expasy.org) [21], and the protein sequences were aligned using the ClustalW, included in the package BioEdit v.7.0.0 (Ibis Biosciences, Carlsbad, CA, USA). The nucleotide sequence data reported in the present study are available in the GenBank, EMBL, and DDBJ databases under the accession numbers OR198481, OR198482, OR291146-OR291149, OR291153-OR291156, OR291418-OR291420, OR291429-OR291431, OR327454, OR327455.

#### Phylogenetic analysis

To determine the phylogenetic relationship of *18S* rRNA gene sequences obtained in this study, we performed a BLAST analysis using the NCBI GenBank database (https://blast.ncbi.nlm.nih.gov/Blast.cgi, accessed on 30 January 2023). Sequences from all continents showing the highest identity to the sequenced samples were selected. At least three sequences from each species were selected for further analysis. The obtained sequences were aligned using the Muscle algorithm in MEGA 11 software [22]. Phylograms were constructed using three different methods, including Maximum Parsimony (MP), Neighbour-joining (NJ), and Maximum Likelihood (ML). Based on their similar topology, the ML method was selected for the final analysis. The Tamura three-parameter model (T92) was used to build the phylogenetic tree, with the removal of unaligned positions (complete deletion), which was determined to be the most suitable based on the lowest Bayesian Information Criterion (BIC) and Corrected Akaike Information Criterion (AICc). To assess the reliability of internal branches, a bootstrapping analysis with 1000 replicates was performed following the methodology described by Tamura et al. [22].

#### Haemoparasite species co-occurrence analysis

To investigate the associations among different haemoparasite species based on binary presence-absence data, we used Yule's Q statistic [18]. Yule's Q measure is formulated for 2 × 2 contingency tables and is mathematically defined as:

$$Yule^{\prime}s{\mkern 1mu} {\mkern 1mu} Q{\mkern 1mu} \, = \, {\mkern 1mu} \frac{ad \, + \, bc}{{ad \, - \, bc}}$$

where *a* and *d* denote the counts of concordant pairs: scenarios in which both species are either present or absent. Conversely, *b* and *c* are the counts of discordant pairs, signifying instances where one species is present while the other is absent. To assess the robustness of these associations, permutation tests were executed, involving repeated randomization of the data set followed by recalculation of Yule’s Q values. This provided a mechanism for deriving *p*-values, thereby quantifying the likelihood that the observed associations occurred by chance.

The range of Yule’s Q values spans from − 1 to + 1, with -1 indicating a perfect negative association, + 1 signifying a perfect positive association, and 0 suggesting no association. In the association matrix derived from Yule’s Q calculations, each element represents the association between respective pairs of parasite species. To improve the precision of subsequent network analyses, a threshold was applied: only associations with an absolute Yule's Q value > 0.3 and a* p*-value > 0.05 were considered.

The co-occurrence network was constructed using the *igraph* package [23] in R, based on the association matrix derived from Yule’s Q. The network was further explored and visualized using Gephi [24], which was also employed for the generation of the final figure. An R script detailing the calculation of Yule's Q and the construction of the co-occurrence network is provided as Additional material (Additional file 1: file S1).

#### Statistical analysis

All obtained data were compiled in Microsoft 365 Excel software (Microsoft Corp., Redmond, WA). The observed prevalence rates and 95% binomial confidence intervals (CI) for each TBP infection and co-infection were determined from microfluidic real-time PCR amplification results. Data analysis tools within Microsoft 365 Excel software (Microsoft Corp., Redmond, WA) were employed for this purpose. Chi-square tests (χ^2^) were used to assess differences in TBP prevalence and co-infection patterns observed between dogs and ticks. Odds ratios (OR) and relative risk (RR), with corresponding 95% CIs, were calculated to evaluate the likelihood of each TBP occurrence among dogs and ticks. Pearson correlation coefficient was used to measure linear correlation of the simultaneous presence of each TBP in dogs and ticks. All statistical analyses were performed with R Statistical Software v4.1.2 [25] using *sjstats* package [26] within the graphical interface R-studio. A clustered heatmap was constructed in R using *ggplot2* and *heatmaply* packages [27, 28]. The heatmap figure was generated based on the correlation matrix derived from Pearson correlation coefficients, providing visual insights into the relationships between TBPs present in both host and vector species. An R script detailing the calculation of Pearson correlation coefficients and the construction of the clustered heatmap is provided as Additional material (Additional file 2: file S2).

## Results

### Tick infestation and health status of dogs

Based on physical examinations, all the dogs were in good health during sample collection, showing no clinical abnormalities, evident health issues, or skin lesions. Furthermore, their body temperatures were within the normal range, and they showed healthy hydration levels. A total of 113 hard ticks were manually detached from the sampled dogs and submitted to the laboratory for identification to species level. All collected tick specimens were identified as *R. sanguineus* s.l., including 59 females and 54 males, through both morphological and molecular analyses. No immature tick stages (i.e. larvae and nymphs) were found among the collected tick specimens. No co-infestation with other tick species was observed on the sampled dogs, indicating a prevalence of *R. sanguineus* s.l. tick species in the studied area.

### Prevalence and diversity of tick-borne pathogen species in dogs and ticks

The internal inhibition control, utilizing specific primers and a probe targeting the *eae* gene to detect *E. coli* strain EDL933 DNA, confirmed the absence of PCR inhibitors in all examined canine blood and tick samples. Additionally, all DNA extraction and non-template controls yielded PCR-negative results. Molecular analysis using a high-throughput microfluidic real-time PCR system revealed that 78.26% (36/46) of the examined dogs tested positive for at least one pathogen. Single infections were observed in 34.78% (16/46) dogs, while co-infections were present in 43.48% (20/46) dogs. A total of eight pathogens were detected with different prevalence. *Hepatozoon canis* was the most common pathogen detected in dogs (43.48%, 20/46), followed by *E. canis* (39.13%, 18/46) and *A. platys* (17.39%, 8/46), while *Ehrlichia* spp. (2.17%, 1/46) and *R. amblyommatis* (2.17%, 1/46) were found in only one dog each (Table 1).

**Table 1:** Vector-borne pathogens detected in blood collected from dogs using microfluidic PCR

Vector-borne pathogen(s)	Total	Prevalence rates (%)	95% CI^a^
Total infected dogs (≥ 1 pathogen)	**36**	**78.26**	**76.50–80.02**
*Hepatozoon canis*	20	43.48	41.37–45.59
*Ehrlichia canis*	18	39.13	37.05–41.21
*Anaplasma platys*	8	17.39	15.78–19.00
*Mycoplasma haemocanis*	5	10.87	9.54–12.20
*Babesia canis* (3 subspecies)	5	10.87	9.54–12.20
*Anaplasma* spp.	4	8.69	7.49–9.89
*Ehrlichia* spp.	1	2.17	1.55–2.79
*Rickettsia amblyommatis*	1	2.17	1.55–2.79
Single infections	**16**	**34.78**	**32.75–36.81**
*Ehrlichia canis*	4	8.69	7.49–9.89
*Hepatozoon canis*	4	8.69	7.49–9,89
*Anaplasma* spp.	2	4.35	3.48–5.22
*Anaplasma platys*	2	4.35	3.48–5.22
*Babesia canis* (3 subspecies)	2	4.35	3.48–5.22
*Ehrlichia* spp.	1	2.17	1.55–2.79
*Mycoplasma haemocanis*	1	2.17	1.55–2.79
Mixed infections	**20**	**43.48**	**41.37–45.59**
Mixed infection with two pathogens	**11**	**23.91**	**22.09–25.73**
*E. canis* + *H. canis*	6	13.04	11.61–14.47
*M. haemocanis* + *H. canis*	2	4.35	3.48–5.22
*Anaplasma* spp. + *R. amblyommatis*	1	2.17	1.55–2.79
*Anaplasma* spp. + *E. canis*	1	2.17	1.55–2.79
*A. platys* + *E. canis*	1	2.17	1.55–2.79
Mixed infection with tree pathogens	**8**	**17.39**	**15.78–19.00**
*A. platys* + *E. canis* + *H. canis*	3	6.52	5.7–7.57
*A. platys* + *H. canis* + *B. canis* (3 subspecies)	2	4.35	3.48–5.22
*E. canis* + *M. haemocanis* + *H. canis*	2	4.35	3.48–5.22
*E. canis* + *H. canis* + *B. canis* (3 subspecies)	1	2.17	1.55–2.79
Non-detected	**11**	**23.91**	**22.09–25.73**

Total prevalence values per category are in bold

^*a*^* 95% confidence interval*

A high-throughput microfluidic real-time PCR system for molecular diagnosis was employed to analyze 46 tick samples, each corresponding to an individual dog. Among them, three tick samples failed to amplify the housekeeping control markers, namely *16S* rRNA and *ITS2* genes, used for tick genus and *R. sanguineus* s.l. species-specific DNA detection, respectively. Consequently, these three samples were excluded from further analyses because of the absence of amplifiable DNA. Overall, 39.53% (17/43) of the examined ticks tested positive for at least one pathogen. Single infections were observed in 30.23% (13/43) ticks, while co-infections were present in 9.30% (4/43) ticks. Five pathogens were detected with different prevalence. *Hepatozoon canis* was the most common pathogen detected in ticks (30.23%, 13/43), followed by *A. platys* (9.30%, 4/43), and *E. canis* (4.65%, 2/43), while *Anaplasma* spp. (2.33%, 1/43) and *R. felis* (2.33%, 1/43) were detected in only one tick each (Table 2). The frequency of single and mixed infections of pathogens in dogs and ticks is presented in Tables 1 and 2, respectively. The statistical analyses showed a significant difference in the overall prevalence of TBPs and their co-infection patterns between dogs and ticks (χ^2^ = 25.326, df = 1, *p*-value = 0.0021).

**Table 2:** Tick-borne pathogens detected in ticks collected from dogs using microfluidic PCR

Vector-borne pathogen(s)	Total	Prevalence rate (%)	95% CI^a^
Total infected ticks (≥ 1 pathogen)	**17**	**39.53**	**37.31–41.76**
*Hepatozoon canis*	13	30.23	28.14–32.33
*Anaplasma platys*	4	9.30	7.98–10.63
*Ehrlichia canis*	2	4.65	3.69–5.61
*Anaplasma* spp.	1	2.33	1.64–3.01
*Rickettsia felis*	1	2.33	1.64–3.01
Single infections	**13**	**30.23**	**28.14–32.33**
*Hepatozoon canis*	9	20.93	19.08–22.78
*Anaplasma platys*	2	4.65	3.69–5.61
*Rickettsia felis*	1	2.33	1.64–3.01
*Anaplasma* spp.	1	2.33	1.64–3.01
Mixed infections	**4**	**9.30**	**7.98–10.63**
Mixed infection with two pathogens	**4**	**9.30**	**7.98–10.63**
*A. platys* + *H. canis*	2	4.65	3.69–5.61
*E. canis* + *H. canis*	2	4.65	3.69–5.61
Non-detected	**24**	**55.81**	**53.55–58.08**

Total prevalence values per category are in bold

^*a*^* 95% confidence interval*

**Fig. 1:**
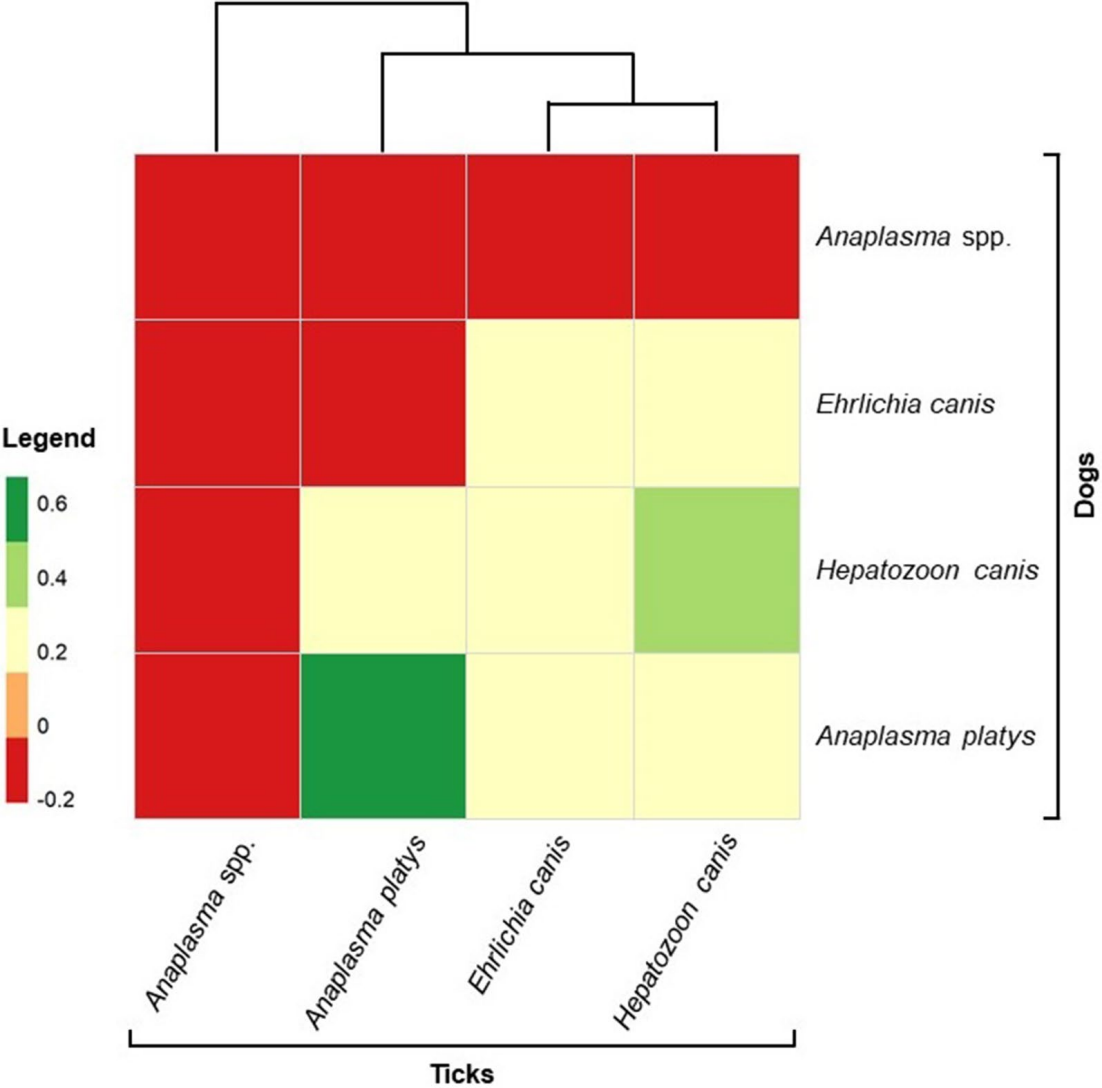
Heatmap illustrating the Pearson correlation coefficients between TBPs presence in dogs and ticks. The heatmap provides insights into the relationships between TBPs detected simultaneously in both host and vector. Correlation values range from − 1 (strong negative correlation) to 1 (strong positive correlation), with 0 indicating no correlation. Shades of green and red represent positive and negative correlations, respectively, while colour intensity reflects the strength of the correlation. The legend on the left side of the heatmap indicates correlation values

The presence of *Anaplasma* spp., *A. platys*, *E. canis*, and *H. canis* was identified in both dogs and ticks. Other pathogens, such as *B. vogeli*, *Ehrlichia* spp., *M. haemocanis*, and *R. amblyommatis* were only detected in dogs, while *R. felis* was solely found in ticks. Overall, the simultaneous occurrence of pathogen infections (i.e. single and mixed infections) in dogs and ticks revealed a statistically non-significant association (χ^2^ = 0.3682, df = 1, *p*-value = 0.544). In contrast, when analyzing each pathogen individually, a significant positive association was observed for the presence of *A. platys* (χ^2^ = 13.824, df = 1, *p*-value < 0.001) and *H. canis* (χ^2^ = 4.237, df = 1, *p*-value = 0.039) in both dogs and ticks (Fig. 1). Notably, the presence of *A. platys* revealed OR and RR values > 1, with OR = 1.53 (95% CI 0.29–8.98) and RR = 1.43 (95% CI 0.31–7.26), indicating a higher likelihood of occurrence in dogs compared to ticks, while the presence of *H. canis* showed OR and RR values < 1, with OR = 0.39 (95% CI 0.12–1.45) and RR = 0.44 (95% CI 0.15–1.25), suggesting a lower likelihood of occurrence in dogs compared to ticks.

### Co-infections and network interactions between pathogens

Among infected dogs (36/46, 78.26%), a significant number showed co-infections (19/36, 52.78%), consisting of 26.08% (12/46) with two microorganisms and 17.39% (8/46) with three microorganisms. Notably, the most prevalent co-infection pattern found was between *E. canis* and *H. canis*, with a frequency of occurrence of 13.04% (6/46). In contrast, among all infected ticks (17/43, 41.46%), a smaller subset displayed co-infections (4/43, 9.3%), characterized exclusively by simultaneous infections involving two pathogen species. Specifically, only two co-infection patterns were observed, including *E. canis* and *H. canis*, as well as *A. platys* and *H. canis*, which both occurred with equal frequency (2/43, 4.65%).

There was non-significant difference (χ^2^ = 23.412, df = 1, *p*-value = 0.103) between the distribution of co-infection patterns in dogs and ticks suggesting an absence of associations between the co-infection patterns and sample types. The co-infection pattern among *E. canis* and *H. canis* was the only one observed in both dogs (6/46, 13.04%) and ticks (2/43, 4.65%) (χ^2^ = 11.917, df = 1, *p*-value = 0.155). Particularly, this co-infection pattern revealed OR and RR values > 1, with OR = 3.32 (95% CI 0.76–16.78) and RR = 3.00 (95% CI 0.74–12.57), which indicate a higher likelihood of occurrence in dogs compared to ticks.

In addition, the statistical analyses of associations among haemoparasites revealed a complexity that is illustrated in the co-occurrence network (Fig. 2). A significant node in this network was identified as *H. canis*, which reflected significant positive association of this species with several other taxa, notably with *A. platys*, *B. vogeli*, *E. canis*, and *M. haemocanis* (*p*-values < 0.05). Further scrutiny of the network unveiled specific species as central players in the co-occurrence dynamics, like *H. canis* was engaged in several positive associations, hinting at a potential facilitative role within the network. Other species, such as *R. felis* and *Ehrlichia* spp., were only involved in negative associations, portraying a potential inhibitory role in the network dynamics. *Mycoplasma haemocanis*, with a similar degree of connectivity, manifested a mixed role with both positive and negative associations, adding a layer of complexity to the parasitic ecosystem dynamics.

**Figure:**
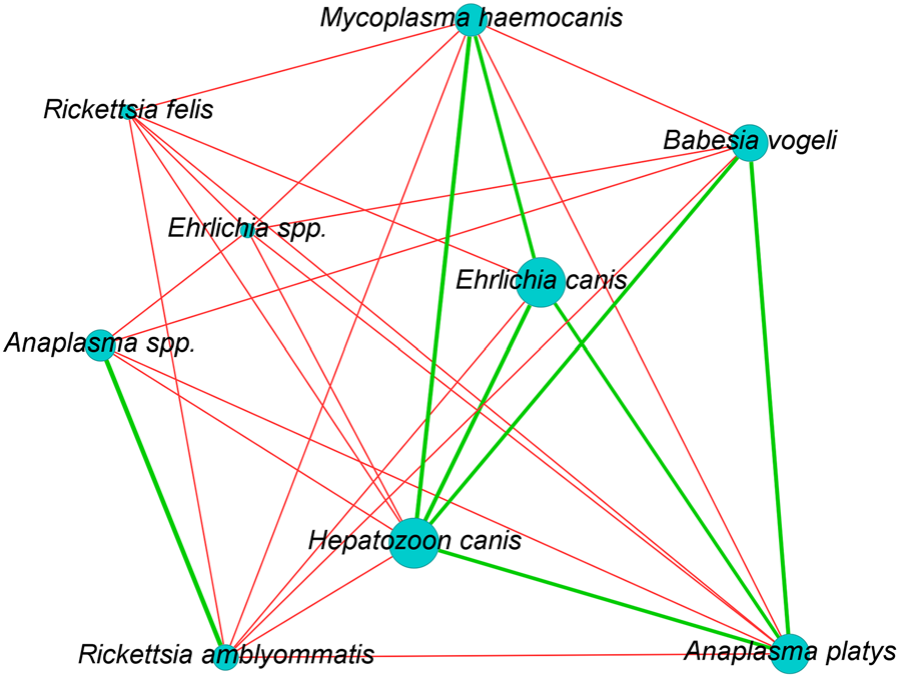
Co-occurrence Network of Haemoparasite Species. The network visualizes significant associations between pairs of parasite species based on Yule's Q statistic. Nodes represent individual parasite species, while edges (connections) represent statistically significant associations. Green edges indicate positive associations, whereas red edges signify negative associations. Only associations with a Yule's Q value > 0.3 and a *p*-value < 0.05 (from permutation tests) are shown. The width of the edges corresponds to the strength of the association

The analysis also highlighted the strongest positive association occurring between *Anaplasma* spp. and *R. amblyommatis*, indicating a potential symbiotic relationship with a weight of 1.0. Conversely, the pair of *A. platys* and *M. haemocanis* demonstrated the strongest negative association, with a weight of – 1.0, perhaps signalling a competitive interaction between these species. Overall, these intricate associations, substantiated by significant *p*-values, paint a detailed picture of the haemoparasite community dynamics, potentially revealing underlying mechanisms of symbiosis and competition that dictate their interactions within the host ecosystem.

### DNA sequencing and phylogenetic diversity of tick-borne pathogens

DNA sequencing was conducted on PCR-positive dogs and tick samples by conventional PCR assays targeting specific genes, including *16S* rRNA gene for *A. platys*, *E. canis*, and *M. haemocanis*; *18S* rRNA gene for *H. canis* and *B. canis*, and the outer membrane protein gene (*ompB*) for *Rickettsia* spp. Among the examined dogs, the BLASTn analysis confirmed the presence of *H. canis* with 100% identity as a previously published sequence from Brazil (KP233215), Pakistan (KU535868), and Portugal (MZ475936). Similarly, the *18S* rRNA gene sequences obtained from *B. canis* (3 subspecies) PCR-positive samples revealed 100% congruence with *Babesia vogeli* (formerly *B. canis vogeli*) sequences previously published from Brazil (KT333456), China (MN067707), and Zambia (LC331058). Furthermore, the sequencing analysis confirmed the presence of *A. platys*, *E. canis*, and *M. haemocanis* in canine blood samples, with their respective sequences showing > 99% identity with previously published sequences from Saint Kitts and Nevis (CP046391), Cuba (MK507008), and Mexico (MN294708), respectively. The identification of *R. amblyommatis* was also confirmed through sequencing, with the obtained *ompB* gene fragment sequence showing 100% identity to known sequences from French Guiana (MT009184).

**Fig. 3:**
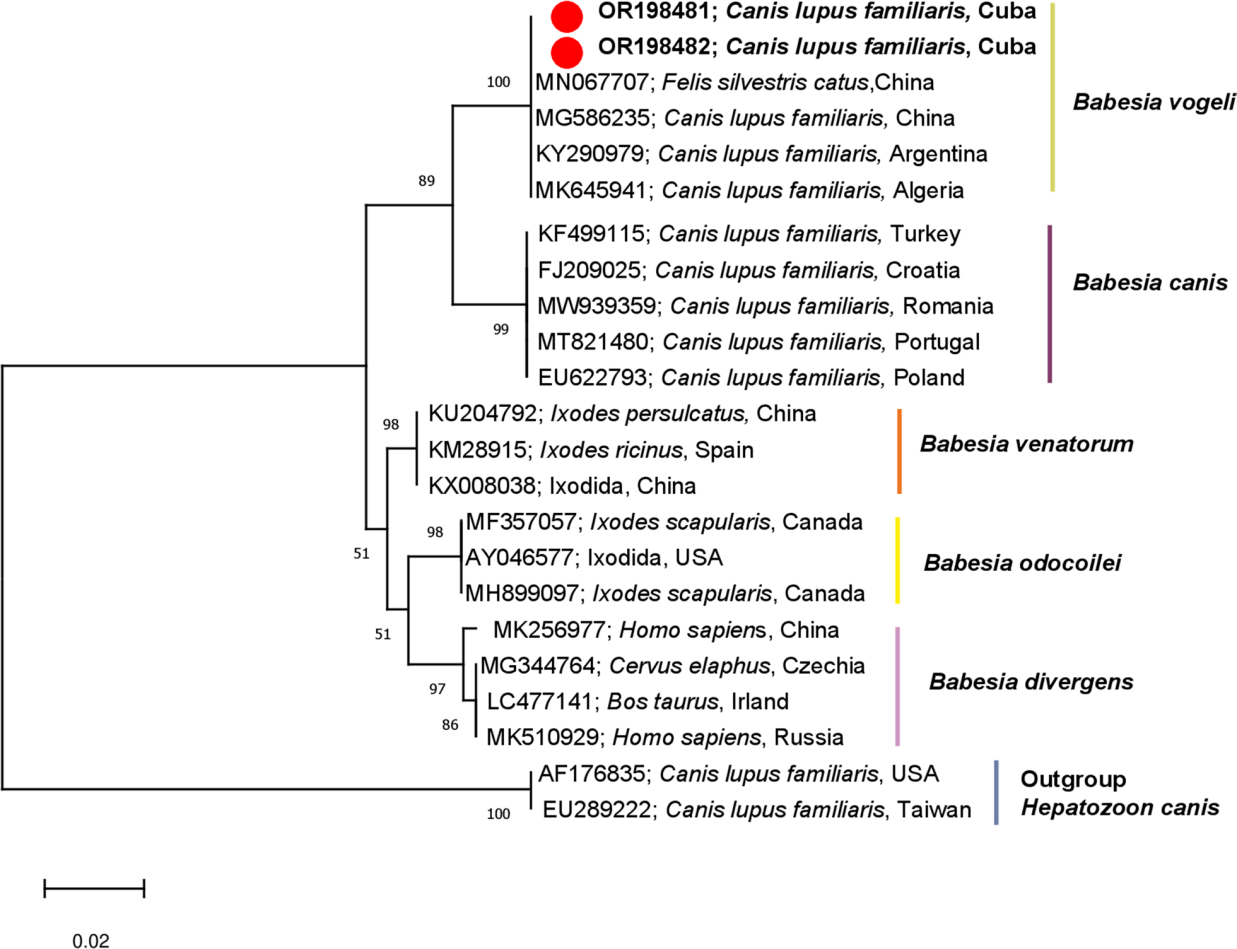
Phylogenetic tree of selected representatives of *Babesia* sp. inferred from 18S rRNA. The evolutionary history was inferred by using the maximum likelihood method and the Tamura 3-parameter model. The analysis contains *Babesia vogeli* 18S rRNA sequences identified in the current study (bold and marked red dots) and GenBank sequences. Accession numbers of sequences, host species, and country of origin are displayed. Bootstrap values are represented as per cent of internal branches (1000 replicates); values < 50 are hidden. The tree is drawn to scale, with branch lengths measured in the number of substitutions per site. This analysis involved 23 nucleotide sequences. There were a total of 410 positions in the final dataset. Sequences AF176835 and EU289222 were used as outgroup

In ticks, the sequences from *A. platys* and *R. felis* PCR-positive samples showed 100% identity with sequences previously deposited in the GenBank from Panama (CP046391) and Sweden (GU182892), respectively. Table 3 provides details of the nucleotide sequences obtained from the genes of TBPs species detected in domestic dog blood samples and *R. sanguineus* s.l. tick species, including the highest percentages of identity with reference strain sequences available in the GenBank database. Unfortunately, sequencing analysis could not be performed for some of the pathogens detected such as *Anaplasma* spp. and *Ehrlichia* spp. because of low cycle threshold (Cq) values of PCR-positive samples. To further investigate the genetic relationships of *B. vogeli*, a phylogenetic analysis was performed based on the *18S* rRNA gene, including sequences from various members of the genus *Babesia*. Interestingly, the novel *B. vogeli* sequences from Cuba showed no major subclustering in the phylogenetic tree (Fig. 3). Moreover, the phylogenetic tree displayed no apparent geographical or host specificity clustering of the *B. vogeli* isolates, suggesting a high level of conservation in the *18S* rRNA genes among canine isolates worldwide.

**Table 3:** Sequencing analyses of the gene fragments amplified for TBPs species detected in blood samples and *Rhipicephalus sanguineus* s.l. tick species collected from domestic dogs (*Canis lupus familiaris*) in Cuba

TBPs species	Host species	Target gene	Query cover (%)	Identity (%)	GenBank	Accession numbers ^a^
*Babesia vogeli*	*Canis lupus familiaris*	*18S* rRNA	99	100	MN067709	OR198481
*Babesia vogeli*	*Canis lupus familiaris*	*18S* rRNA	100	100	MN067707	OR198482
*Anaplasma platys*	*Canis lupus familiaris*	*16S* rRNA	100	99.82	CP046391	OR291146
*Anaplasma platys*	*Canis lupus familiaris*	*16S* rRNA	100	100	MN630836	OR291147
*Anaplasma platys*	*Canis lupus familiaris*	*16S* rRNA	100	100	MN630836	OR291148
*Anaplasma platys*	*Rhipicephalus sanguineus*	*16S* rRNA	99	100	MN630836	OR291149
*Ehrlichia canis*	*Canis lupus familiaris*	*16S* rRNA	100	100	MK507008	OR291153
*Ehrlichia canis*	*Canis lupus familiaris*	*16S* rRNA	99	99.82	MK507008	OR291154
*Ehrlichia canis*	*Canis lupus familiaris*	*16S* rRNA	99	99.66	MK507008	OR291155
*Ehrlichia canis*	*Canis lupus familiaris*	*16S* rRNA	100	100	MK507008	OR291156
*Hepatozoon canis*	*Canis lupus familiaris*	*18S* rRNA	100	100	KU535868	OR291418
*Hepatozoon canis*	*Canis lupus familiaris*	*18S* rRNA	100	100	LC331053	OR291419
*Hepatozoon canis*	*Canis lupus familiaris*	*18S* rRNA	100	100	MT433125	OR291420
*Mycoplasma haemocanis*	*Canis lupus familiaris*	*16S* rRNA	100	98.98	MK230032	OR291429
*Mycoplasma haemocanis*	*Canis lupus familiaris*	*16S* rRNA	99	98.12	MK239932	OR291430
*Mycoplasma haemocanis*	*Canis lupus familiaris*	*16S* rRNA	100	98.84	MK239932	OR291431
*Rickettsia amblyommatis*	*Canis lupus familiaris*	*ompB*	100	100	MT009184	OR327454
*Rickettsia felis*	*Rhipicephalus sanguineus*	*ompB*	100	99.26	ON053303	OR327455

The table presents the highest percentages of identity with reference strain sequences available in the GenBank database

^a^Accession numbers of sequences submitted in this study

## Discussion

To the best of the authors' knowledge, the present study represents the first comprehensive investigation to utilize a high-throughput microfluidic real-time PCR system for large-scale screening of TBPs in both dogs and ticks from Cuba. Notably, we used co-occurrence network analysis to study interactions between identified pathogens. Overall, the DNA of TBPs such as bacteria and protozoan belonging to six genera—*Anaplasma*, *Babesia*, *Ehrlichia*, *Hepatozoon*, *Mycoplasma*, and *Rickettsia*—was identified in both the examined dogs and ticks. The dog samples revealed the presence of *H. canis*, *E. canis*, *A. platys*, *M. haemocanis*, *R. amblyommatis*, and *B. vogeli*, whereas in the tick samples, *H. canis*, *A. platys*, and *R. felis* were detected.

First and foremost, our identification of a diverse range of TBPs in both hosts underscores the complexity of the interactions between these pathogens and their vectors [29]. Previous studies conducted in the Caribbean region have detected various TBPs in dogs, including *A. platys*, *B. vogeli*, *E. canis*, *H. canis*, and *M. haemocanis*, for which *R. sanguineus* s.l. tick species have been identified as the main arthropod vector [30, 31]. The high prevalence of *H. canis*, *E. canis*, and *A. platys* described in this study is consistent with previous studies conducted in Cuba, which raises concerns given they are well-known causative agents of canine TBDs, and suggests the ongoing risk these pathogens pose to the canine population [9, 10]. Moreover, the presence of *E. canis* and *A. platys* highlights the necessity of vigilance and awareness among dog owners concerning the risks of TBP infection to their pets and the potential zoonotic transmission to humans [32].

The co-occurrence network analysis undertaken in this study has unveiled a new dimension to our understanding of the intricate relationships between these pathogens. The significant positive and negative associations observed between various pairs of pathogens suggest a complex web of interactions that might be facilitating or inhibiting their coexistence within the host organisms. Particularly, *H. canis* emerged as a focal point in this web, establishing a series of significant associations with other pathogens, indicating its pivotal role in the co-infection dynamics [33]. This finding hints at a potentially higher adaptability of *H. canis* in establishing infections in conjunction with a variety of other pathogens, thereby possibly influencing the infection patterns observed in this study [34]. Understanding these associations further could be vital in developing targeted interventions to manage these co-infections effectively [35, 36].

The detection of *R. felis* in a tick sample highlights the potential role of *R. sanguineus* s.l. as a vector of a spotted fever group (SFG) rickettsia within Cuba. This finding is consistent with previous studies worldwide that have reported the presence of *R. felis* in dogs, *R. sanguineus* s.l. ticks collected from dogs, and humans [29]. In Cuba, *R. felis* has been described in both stray and shelter dogs [9], as well as in *Dermacentor nitens* tick species collected from horses [37]. The diverse clinical manifestations attributed to *R. felis* infection in humans and animals emphasize the significance of monitoring its presence within tick populations [38]. Additionally, it is noteworthy that the *R. felis* strain detected in the present study shared 100% identity with a strain detected in Sweden associated with subacute meningitis in humans [37]. Nevertheless, further research is warranted to fully elucidate the role of *R. sanguineus* s.l. as a competent vector of *R. felis* among dog populations in Cuba.

While the presence of *B. vogeli* has been previously reported in *R. sanguineus* s.l. ticks from Cuba by Navarrete and Cordeiro [13], this study represents the first molecular report of *B. vogeli* infections in domestic dogs from Cuba. The phylogenetic analysis conducted for *B. vogeli* aimed to determine the relationship between the obtained sequences herein and those from different geographic regions worldwide. This analysis revealed that the examined sequences clustered alongside other *B. vogeli 18S* rRNA gene sequences reported from Asia, Africa, and South America. The lack of major subclustering of *B. vogeli* sequences observed in the phylogenetic tree suggests a high degree of genetic conservation of the *18S* rRNA gene among *B. vogeli* isolates across different geographical regions and host populations. This level of genetic conservation indicates the stability of this pathogen and its potential to adapt to a wide range of hosts and vectors [39]. Further investigation into the genetic mechanisms behind this genetic conservation could provide key aspects of the evolutionary and ecological dynamics of *B. vogeli*.

The significant differences in prevalence and co-infection patterns between dogs and ticks are indicative of distinct host–pathogen interactions. While this study does not provide a comprehensive explanation for these differences, they could be influenced by factors such as host immune responses, vector feeding behaviours, and ecological variations [40]. Additionally, the co-occurrence network analysis sheds light on the possible synergistic or antagonistic interactions occurring between different pathogens within the same hosts [41]. This analysis showcases an intricate web of inter-species associations, which suggests that certain pathogens might interact within hosts or vectors, with potential implications for disease progression and transmission dynamics [42]. Notably, the positive association between *Anaplasma* spp. and *R. amblyommatis* may signal a symbiotic relationship, facilitating their co-existence within the hosts [43]; however, given the limited number of positive samples for *R. amblyommatis* (only one sample) in our study, it is essential to approach the potential association with *Anaplasma* spp. with caution. In contrast, the negative association observed between *A. platys* and *M. haemocanis* might indicate a competitive interaction, potentially influencing their prevalence in the host populations [44]. These findings underscore the necessity for a deeper exploration into the mechanisms driving these interactions, which might hold the key to understanding the broader dynamics of TBDs in the region [45].

The findings described above are particularly interesting because of the potential public health implications of co-infections, since simultaneous infections with different TBPs are commonly reported in both humans and companion animals worldwide [46]. The presence of multiple pathogens within a single tick or vertebrate host is common, yet our understanding of their interactions remains limited [41]. The occurrence of TBP co-infections underscores the complex nature of TBDs and highlights several significant implications for disease dynamics, clinical outcomes, and transmission patterns [47]. These co-infections can substantially challenge diagnostic approaches. For instance, conventional molecular diagnostic assays like end-point PCR often target a single pathogen, which might lead to underestimation of co-infection rates [48]. In this context, the utilization of a microfluidic real-time PCR system for the diagnosis of TBPs in the present study is particularly noteworthy. The use of this innovative approach in large-scale epidemiological studies of TBPs has enabled rapid, sensitive, specific, and simultaneous detection of a wide array of pathogens, providing a more comprehensive assessment of pathogens species diversity and co-infection patterns [49].

The presence of multiple TBPs can elicit complex immune responses within the host, leading to different types of adaptive immune mechanisms in hosts such as antibody production and cytotoxic and/or T helper cell responses [50]. These immune effectors contribute to the immune responses of the host, particularly in the later stages, providing long-lasting protection. Tick-borne pathogen co-infections can interact with each other and the tick's microbiome, affecting the fitness, virulence, infectivity, and transmission of individual pathogens [40]. In addition, the presence of multiple TBPs can modulate immune system activation, which could either enhance or suppress the host's ability to clear infections, consequently influencing the outcome of infection and disease progression [51]. These co-infections can lead to more severe clinical presentations, atypical symptoms, prolonged disease duration, and thus complicate diagnosis and therapeutic treatment strategies. The occurrence of mixed TBP infections can potentially result in synergistic interactions, exacerbating disease symptoms and potentially leading to more severe outcomes [40]. Therefore, clinicians should consider the possibility of co-infections when diagnosing TBDs, as treatment strategies may need to be adjusted accordingly [52].

## Conclusions

This study provides valuable insights into the prevalence, diversity, and co-infection patterns of TBPs in dogs and ticks from Cuba. The findings highlight the high prevalence of TBPs in the region, with *R. sanguineus* s.l. identified as the primary vector for transmitting various TBDs. The study emphasizes the importance of tick surveillance and raises awareness among dog owners about the risks associated with TBDs, particularly in areas where *R. sanguineus* s.l. is prevalent. The use of microfluidic-based high-throughput real-time PCR systems enabled rapid and sensitive detection of TBPs, facilitating large-scale epidemiological investigations. Molecular confirmation of specific pathogens through DNA sequencing and phylogenetic analysis provided valuable insights into their identity and relatedness. The co-infections and network interactions observed in both dogs and ticks highlight the complexity of TBPs ecology and the need for comprehensive diagnostic and management strategies. These findings contribute to our understanding of the intricate relationships between TBPs and their hosts. This research not only advances our comprehension of the complex ecological dynamics within ticks but also offers a profound understanding of the potential implications for public health and animal welfare in Cuba.

### Supplementary Information

**Additional file 1** R script detailing the calculation of Yule's Q and the construction of the co-occurrence network.

**Additional file 2** R script detailing the calculation of Pearson correlation coefficients and the construction of the clustered heatmap.

**Additional file 3**:** Table 1**. List of primer/probe sets used in the BioMark™ real-time PCR system.

**Additional file 4**:** Table 2**. Primer pairs and PCR conditions used for validation of microfluidic real-time PCR results.

## Data Availability

DNA sequences obtained in this study were submitted to GenBank (https://www.ncbi.nlm.nih.gov) and accession numbers were assigned (OR198481, OR198482, OR291146-OR291149, OR291153-OR291156, OR291418-OR291420, OR291429-OR291431, OR327454, OR327455).

## References

[1] Boulanger N, Boyer P, Talagrand-Reboul E, Hansmann Y (2019). Ticks and tick-borne diseases. Med Mal Infect.

[2] de la Fuente J, Estrada-Peña A, Venzal JM, Kocan KM, Sonenshine DE (2008). Overview: ticks as vectors of pathogens that cause disease in humans and animals. Front Biosci.

[3] Moutailler S, Valiente Moro C, Vaumourin E, Michelet L, Tran FH, Devillers E (2016). Co-infection of ticks: The rule rather than the exception. PLoS Negl Trop Dis.

[4] Namina A, Capligina V, Seleznova M, Krumins R, Aleinikova D, Kivrane A (2019). Tick-borne pathogens in ticks collected from dogs, Latvia, 2011–2016. BMC Vet Res.

[5] Herrmann JA, Dahm NM, Ruiz MO, Brown WM (2014). Temporal and spatial distribution of tick-borne disease cases among humans and canines in Illinois (2000–2009). Environ Health Insights.

[6] Dantas-Torres F, Latrofa MS, Annoscia G, Giannelli A, Parisi A, Otranto D (2013). Morphological and genetic diversity of *Rhipicephalus sanguineus* sensu lato from the New and Old Worlds. Parasit Vectors.

[7] Dantas-Torres F (2010). Biology and ecology of the brown dog tick, *Rhipicephalus sanguineus*. Parasit Vectors.

[8] Barros-Battesti DM, Hernández MR, Famadas KM, Onofrio VC, Beati L, Guglielmone AA (2009). The ixodid ticks (Acari: *Ixodidae*) of Cuba. Syst Appl Acarol.

[9] Diaz-Sanchez AA, Corona-Gonzalez B, Meli ML, Roblejo-Arias L, Fonseca-Rodriguez O, Perez Castillo A (2020). Molecular diagnosis, prevalence and importance of zoonotic vector-borne pathogens in cuban shelter dogs-a preliminary study. Pathogens..

[10] Diaz-Sanchez AA, Hofmann-Lehmann R, Meli ML, Roblejo-Arias L, Fonseca-Rodriguez O, Castillo AP (2021). Molecular detection and characterization of *Hepatozoon canis* in stray dogs from Cuba. Parasitol Int.

[11] Roblejo-Arias L, Diaz-Sanchez AA, Corona-Gonzalez B, Meli ML, Fonseca-Rodriguez O, Rodriguez-Mirabal E (2022). First molecular evidence of *Mycoplasma haemocanis* and '*Candidatus* Mycoplasma haematoparvum' infections and its association with epidemiological factors in dogs from Cuba. Acta Trop.

[12] Silva CBD, Santos HA, Navarrete MG, Ribeiro C, Gonzalez BC, Zaldivar MF (2016). Molecular detection and characterization of *Anaplasma platys* in dogs and ticks in Cuba. Ticks Tick Borne Dis.

[13] Navarrete MG, Cordeiro MD, da Silva CB, Pires MS, Ribeiro CCDU, Cruz AC (2016). Molecular detection of *Ehrlichia canis* and *Babesia canis vogeli* in *Rhipicephalus sanguineus* sensu lato ticks from Cuba. Rev Bras Med Vet.

[14] Noda AA, Rodriguez I, Miranda J, Mattar S, Cabezas-Cruz A (2016). First report of spotted fever group *Rickettsia* in cuba. Ticks Tick Borne Dis..

[15] Michelet L, Delannoy S, Devillers E, Umhang G, Aspan A, Juremalm M (2014). High-throughput screening of tick-borne pathogens in Europe. Front Cell Infect Microbiol.

[16] Banovic P, Diaz-Sanchez AA, Galon C, Simin V, Mijatovic D, Obregon D (2021). Humans infested with *Ixodes ricinus* are exposed to a diverse array of tick-borne pathogens in Serbia. Ticks Tick Borne Dis.

[17] Ghafar A, Cabezas-Cruz A, Galon C, Obregon D, Gasser RB, Moutailler S (2020). Bovine ticks harbour a diverse array of microorganisms in Pakistan. Parasit Vectors.

[18] Lipsitz SR, Fitzmaurice G (1994). An extension of Yule's Q to multivariate binary data. Biometrics.

[19] Walker JB, Keirans JE, Horak IG (2005). The genus *Rhipicephalus* (Acari, *Ixodidae*): a guide to the brown ticks of the world.

[20] Grech-Angelini S, Stachurski F, Vayssier-Taussat M, Devillers E, Casabianca F, Lancelot R, Uilenberg G, Moutailler S (2020). Tick-borne pathogens in ticks (Acari: *Ixodidae*) collected from various domestic and wild hosts in Corsica (France), a Mediterranean island environment. Transbound Emerg Dis.

[21] Altschul SF, Gish W, Miller W, Myers EW, Lipman DJ (1990). Basic local alignment search tool. J Mol Biol.

[22] Duvaud S, Gabella C, Lisacek F, Stockinger H, Ioannidis V, Durinx C (2021). Expasy, the swiss bioinformatics resource portal, as designed by its users. Nucleic Acids Res.

[23] Tamura K, Stecher G, Kumar S (2021). MEGA11: molecular evolutionary genetics analysis version 11. Mol Biol Evol.

[24] Nepusz G, Csárdi G (2006). The igraph software package for complex network research. Complex Syst.

[25] Bastian M, Heymann S, Jacomy M (2009). Gephi: an open source software for exploring and manipulating networks. ICWSM.

[26] Team RC (2021). R: A language and environment for statistical computing.

[27] Lüdecke D. sjstats: Statistical Functions for Regression Models (Version 0.18.2). 2022.

[28] Tollefson M, Tollefson M (2021). Graphics with the *ggplot2* Package: An introduction. Visualizing data in R 4: Graphics using the base, graphics, stats, *ggplot2* Packages.

[29] Galili T, O'Callaghan A, Sidi J, Sievert C (2018). *heatmaply*: an R package for creating interactive cluster heatmaps for online publishing. Bioinformatics.

[30] Banovic P, Diaz-Sanchez AA, Galon C, Foucault-Simonin A, Simin V, Mijatovic D (2021). A One Health approach to study the circulation of tick-borne pathogens: a preliminary study. One Health.

[31] Loftis AD, Kelly PJ, Freeman MD, Fitzharris S, Beeler-Marfisi J, Wang C (2013). Tick-borne pathogens and disease in dogs on St Kitts West Indies. Vet Parasitol.

[32] Starkey LA, Newton K, Brunker J, Crowdis K, Edourad EJP, Meneus P (2016). Prevalence of vector-borne pathogens in dogs from Haiti. Vet Parasitol.

[33] Reller ME, Dumler JS (2015). *Ehrlichia*, *Anaplasma*, and related intracellular bacteria. Manual Clin Microbiol.

[34] Cabezas-Cruz A, Allain E, Ahmad AS, Saeed MA, Rashid I, Ashraf K (2019). Low genetic diversity of *Ehrlichia canis* associated with high co-infection rates in *Rhipicephalus sanguineus* (s.l.). Parasit Vectors..

[35] Kuručki M, Tomanović S, Sukara R, Ćirović D (2022). High prevalence and genetic variability of Hepatozoon canis in Grey Wolf (*Canis lupus* L. 1758) population in Serbia. Animals.

[36] Baxarias M, Alvarez-Fernandez A, Martinez-Orellana P, Montserrat-Sangra S, Ordeix L, Rojas A (2018). Does co-infection with vector-borne pathogens play a role in clinical canine leishmaniosis?. Parasit Vectors.

[37] Zhang SX, Zhou YM, Xu W, Tian LG, Chen JX, Chen SH (2016). Impact of co-infections with enteric pathogens on children suffering from acute diarrhea in southwest China. Infect Dis Poverty.

[38] Diaz-Sanchez AA, Chilton NB, Roblejo-Arias L, Fonseca-Rodriguez O, Marrero-Perera R, Diyes CP (2021). Molecular detection and identification of spotted fever group rickettsiae in ticks collected from horses in Cuba. Med Vet Entomol.

[39] Perez-Osorio CE, Zavala-Velazquez JE, Arias Leon JJ, Zavala-Castro JE (2008). *Rickettsia felis* as emergent global threat for humans. Emerging Infect Dis.

[40] Lindblom A, Severinson K, Nilsson K (2010). *Rickettsia felis* infection in Sweden: report of two cases with subacute meningitis and review of the literature. Scand J Infect Dis.

[41] Nehra AK, Kumari A, Moudgil AD, Vohra S (2021). Phylogenetic analysis, genetic diversity and geographical distribution of *Babesia caballi* based on *18S* rRNA gene. Ticks Tick Borne Dis.

[42] Gomez-Chamorro A, Hodzic A, King KC, Cabezas-Cruz A (2021). Ecological and evolutionary perspectives on tick-borne pathogen co-infections. Curr Res Parasitol Vector Borne Dis.

[43] Cutler SJ, Vayssier-Taussat M, Estrada-Peña A, Potkonjak A, Mihalca AD, Zeller H (2021). Tick-borne diseases and co-infection: current considerations. Ticks Tick Borne Dis.

[44] Chicana B, Couper LI, Kwan JY, Tahiraj E, Swei A (2019). Comparative microbiome profiles of sympatric tick species from the Far-Western United States. Insects.

[45] Adegoke A, Kumar D, Bobo C, Rashid MI, Durrani AZ, Sajid MS (2020). Tick-borne pathogens shape the native microbiome within tick vectors. Microorganisms.

[46] Genne D, Sarr A, Gomez-Chamorro A, Durand J, Cayol C, Rais O (1890). Competition between strains of *Borrelia afzelii* inside the rodent host and the tick vector. Proc Biol Sci.

[47] de la Fuente J, Antunes S, Bonnet S, Cabezas-Cruz A, Domingos AG, Estrada-Pena A (2017). Tick-pathogen interactions and vector competence: identification of molecular drivers for tick-borne diseases. Front Cell Infect Microbiol.

[48] Karshima SN, Karshima MN, Ahmed MI (2022). Infection rates, species diversity, and distribution of zoonotic *Babesia* parasites in ticks: a global systematic review and meta-analysis. Parasitol Res.

[49] Salata C, Moutailler S, Attoui H, Zweygarth E, Decker L, Bell-Sakyi L (2021). How relevant are in vitro culture models for study of tick-pathogen interactions?. Pathog Glob Health.

[50] Tokarz R, Lipkin WI (2021). Discovery and surveillance of tick-borne pathogens. J Med Entomol.

[51] Gondard M, Delannoy S, Pinarello V, Aprelon R, Devillers E, Galon C (2020). Upscaling the surveillance of tick-borne pathogens in the French Caribbean Islands. Pathogens..

[52] Torina A, Blanda V, Villari S, Piazza A, La Russa F, Grippi F (2020). Immune response to tick-borne haemoparasites: Host adaptive immune response mechanisms as potential targets for therapies and vaccines. Int J Med Mol Sci..

[53] Karim S, Kumar D, Budachetri K (2021). Recent advances in understanding tick and rickettsiae interactions. Parasite Immunol.

[54] Sanchez E, Vannier E, Wormser GP, Hu LT (2016). Diagnosis, treatment, and prevention of lyme disease, human granulocytic anaplasmosis, and babesiosis: a review. JAMA.

