# Supplementary material for: Microfluidic PCR and network analysis reveals complex tick-borne pathogen interactions in the tropics
Source: Parasit Vectors. 2024 Jan 4;17:5. doi: 10.1186/s13071-023-06098-0 (PMC10765916; doi:10.1186/s13071-023-06098-0)
Supplement: Supplementary file 3 — Additional file 3: Table 1. List of primer/probe sets used in the BioMark™ real-time PCR system. [file 13071_2023_6098_MOESM3_ESM.doc]

**Supplementary Table 1.** List of primer/probe sets used in the BioMark™ real-time PCR system.

| **Microorganisms** | **Target** | **Design Name** | **Sequence (5′ à 3′)** | **Length (bp)** |
| --- | --- | --- | --- | --- |
| *Mycoplasma* spp. | *16S rRNA* | Myc_spp_16S_F | GTGACGGCTAACTATGTGCC | 77 |
|  |  | Myc_spp_16S_R | CGGAATTATTGGGCGTAAAGC |  |
|  |  | Myc_spp_16S_P | AGCAGCTGCGGTAATACATAGGTCGC |  |
| *Mycoplasma haemofelis* | *dnak* | Myc_hfe_dnaK_F | TTGGCTACTTCTGGTGATAACC | 90 |
|  |  | Myc_hfe_dnaK_R | GTGTTCCTTCTTGATTTCCTCTA |  |
|  |  | Myc_hfe_dnaK_P | ATTTGGGTGGTGACGATTGGGATCAAGC |  |
| *Mycoplasma ovis* | *rnpB* | Myc_ov_rnpB_F | GACTCAACGACTAGTTCTACTAG | 106 |
|  |  | Myc_ov_rnpB_R | CTATAAAGCTCGTCTCTGTGG |  |
|  |  | Myc_ov_rnpB_P | TTGATGGCGGAGAACTTTTCTAAATAAGCC |  |
| *‘Candidatus Mycoplasma haematoparvum’* | *(CMhp) rap1* | Myc_hpa_16S_F | CGAAGAGGGCTTGCCCTC | 85 |
|  |  | Myc_hpa_16S_R | CCTTTCGGATTGCTATTCTCC |  |
|  |  | Myc_hpa_16S_P | TTAGTGGCGAACGGGCGAGTAACGC |  |
| *Borrelia* spp. | *23SrRNA* | Bo_bu_sl_23S_F | GAGTCTTAAAAGGGCGATTTAGT | 73 |
|  |  | Bo_bu_sl_23S_R | CTTCAGCCTGGCCATAAATAG |  |
|  |  | Bo_bu_sl_23S_P | AGATGTGGTAGACCCGAAGCCGAGT |  |
| *Borrelia miyamotoi* | *glpQ* | B_miy_glpQ_F | CACGACCCAGAAATTGACACA | 94 |
|  |  | B_miy_glpQ_R | GTGTGAAGTCAGTGGCGTAAT |  |
|  |  | B_miy_glpQ_P | TCGTCCGTTTTCTCTAGCTCGATTGGG |  |
| *Anaplasma* spp. | *16S rRNA* | Ana_spp_16S_F | CTTAGGGTTGTAAAACTCTTTCAG | 160 |
|  |  | Ana_spp_16S_R | CTTTAACTTACCAAACCGCCTAC |  |
|  |  | Ana_spp_16S_P | ATGCCCTTTACGCCCAATAATTCCGAACA |  |
| *Anaplasma marginale* | *msp*1b | An_ma_msp1_F | CAGGCTTCAAGCGTACAGTG | 85 |
|  |  | An_ma_msp1_R | GATATCTGTGCCTGGCCTTC |  |
|  |  | An_ma_msp1_P | ATGAAAGCCTGGAGATGTTAGACCGAG |  |
| *Anaplasma platys* | *gro*EL | An_pl_groEL_F | TTCTGCCGATCCTTGAAAACG | 75 |
|  |  | An_pl_groEL_R | CTTCTCCTTCTACATCCTCAG |  |
|  |  | An_pl_groEL_P | TTGCTAGATCCGGCAGGCCTCTGC |  |
| *Anaplasma phagocytophilum* | *msp*2 | An_ph_msp2_F | GCTATGGAAGGCAGTGTTGG | 77 |
|  |  | An_ph_msp2_R | GTCTTGAAGCGCTCGTAACC |  |
|  |  | An_ph_msp2_P | AATCTCAAGCTCAACCCTGGCACCAC |  |
| *Anaplasma bovis* | *groEL* | An_bov_groEL_F | GGGAGATAGTACACATCCTTG | 73 |
|  |  | An_bov_groEL_R | CTGATAGCTACAGTTAAGCCC |  |
|  |  | An_bov_groEL_P | AGGTGCTGTTGGATGTACTGCTGGACC |  |
| *Ehrlichia* spp. | *16S rRNA* | Eh_spp_16S_F | GCAACGCGAAAAACCTTACCA | 98 |
|  |  | Eh_spp_16S_R | AGCCATGCAGCACCTGTGT 98 |  |
|  |  | Eh_spp_16S_P | AAGGTCCAGCCAAACTGACTCTTCCG |  |
| *Ehrlichia ewingi* | *dsb* | Eh_ew_dsb_F | CAATACTTGGAGAAGCATCATTG | 111 |
|  |  | Eh_ew_dsb_R | TTGCTTATGGCTTAATGCTGCAT |  |
|  |  | Eh_ew_dsb_P | AAAGCAGTACGTGCAGCATTGGCTGTA |  |
| *Ehrlichia chaffeensis* | *dsb* | Eh_ch_dsb_F | TATTGCTAATTACCCTCAAAAAGTC | 117 |
|  |  | Eh_ch_dsb_R | GAGCTATCCTCAAGTTCAGATTT |  |
|  |  | Eh_ch_dsb_P | ATTGACCTCCTAACTAGAGGGCAAGCA |  |
| *Ehrlichia canis* | *dsb* | Eh_ca_dsb_F | AATACTTGGTGAGTCTTCACTCA | 110 |
|  |  | Eh_ca_dsb_R | GTTGCTTGTAATGTAGTGCTGC |  |
|  |  | Eh_ca_dsb_P | AAGTTGCCCAAGCAGCACTAGCTGTAC |  |
| *Rickettsia* spp. | *gltA* | Ri_spp_gltA_F | GTCGCAAATGTTCACGGTACTT | 78 |
|  |  | Ri_spp_gltA_R | TCTTCGTGCATTTCTTTCCATTG 78 |  |
|  |  | Ri_spp_gltA_P | TGCAATAGCAAGAACCGTAGGCTGGATG |  |
| *Neoehrlichia mikurensis* | *gro*EL | Nm_groEL_F | AGAGACATCATTCGCATTTTGGA | 96 |
|  |  | Nm_groEL_R | TTCCGGTGTACCATAAGGCTT |  |
|  |  | Nm_groEL_P | AGATGCTGTTGGATGTACTGCTGGACC |  |
| *Rickettsia conorii* | *23S-5S ITS* | Ri_co_ITS_F | CTCACAAAGTTATCAGGTTAAATAG | 118 |
|  |  | Ri_co_ITS_R | CGATACTCAGCAAAATAATTCTCG |  |
|  |  | Ri_co_ITS_P | CTGGATATCGTGGCAGGGCTACAGTAT |  |
| *Rickettsia slovaca* | *23S-5S ITS* | Ri_sl_ITS_F | GTATCTACTCACAAAGTTATCAGG | 138 |
|  |  | Ri_sl_ITS_R | CTTAACTTTTACTACAATACTCAGC |  |
|  |  | Ri_sl_ITS_P | TAATTTTCGCTGGATATCGTGGCAGGG |  |
| *Rickettsia massiliae* | *23S-5S ITS* | Ri_ma_ITS_F | GTTATTGCATCACTAATGTTATACTG | 128 |
|  |  | Ri_ma_ITS_R | GTTAATGTTGTTGCACGACTCAA |  |
|  |  | Ri_ma_ITS_P | TAGCCCCGCCACGATATCTAGCAAAAA |  |
| *Rickettsia helvetica* | *23S-5S ITS* | Ri_he_ITS_F | AGAACCGTAGCGTACACTTAG | 79 |
|  |  | Ri_he_ITS_R | GAAAACCCTACTTCTAGGGGT |  |
|  |  | Ri_he_ITS_P | TACGTGAGGATTTGAGTACCGGATCGA |  |
| *Rickettsia aeschlimannii* | *ITS* | Rick_aesch_ITS_F | CTCACAAAGTTATCAGGTTAAATAG | 134 |
|  |  | Rick_aesch_ITS_R | CTTAACTTTTACTACGATACTTAGCA |  |
|  |  | Rick_aesch_ITS_P | TAATTTTTGCTGGATATCGTGGCGGGG |  |
| *Rickettsia felis* | *orfB* | Ri_fel_orfB_F | ACCCTTTTCGTAACGCTTTGC | 163 |
|  |  | Ri_fel_orfB_R | TATACTTAATGCTGGGCTAAACC |  |
|  |  | Ri_fel_orfB_P | AGGGAAACCTGGACTCCATATTCAAAAGAG |  |
| *Rickettsia rickettsii* | *23S-5S ITS* | Ri_ri_ITS_F | TCTACTCACAAAGTTATCAGGTTAA | 124 |
|  |  | Ri_ri_ITS_R | CCTACGATACTCAGCAAAATAATTT |  |
|  |  | Ri_ri_ITS_P | TCGCTGGATATCGTTGCAGGACTACAG |  |
| *Bartonella* spp. | *ssrA* | Bart_spp_ssrA_F | CGTTATCGGGCTAAATGAGTAG | 118 |
|  |  | Bart_spp_ssrA_R | ACCCCGCTTAAACCTGCGA |  |
|  |  | Bart_spp_ssrA_P | TTGCAAATGACAACTATGCGGAAGCACGTC |  |
| *Bartonella henselae* | *pap31* | Bar_he_pap_F | CCGCTGATCGCATTATGCCT | 107 |
|  |  | Bar_he_pap_R | AGCGATTTCTGCATCATCTGCT |  |
|  |  | Bar_he_pap_P | ATGTTGCTGGTGGTGTTTCCTATGCAC |  |
| *Francisella tularensis* | *tul4* | Fr_tu_tul4_F | ACCCACAAGGAAGTGTAAGATTA | 76 |
|  |  | Fr_tu_tul4_R | GTAATTGGGAAGCTTGTATCATG |  |
|  |  | Fr_tu_tul4_P | AATGGCAGGCTCCAGAAGGTTCTAAGT |  |
| *Francisella-like endosymbionts* | *fopA* | Fr_tu_fopA_F | GGCAAATCTAGCAGGTCAAGC | 91 |
|  |  | Fr_tu_fopA_R | CAACACTTGCTTGAACATTTCTAG |  |
|  |  | Fr_tu_fopA_P | AACAGGTGCTTGGGATGTGGGTGGTG |  |
| *Coxiella-like endosymbionts* | *IS1111* | Co_bu_IS_F | TGGAGGAGCGAACCATTGGT | 86 |
|  |  | Co_bu_IS_R | CATACGGTTTGACGTGCTGC |  |
|  |  | Co_bu_IS_P | ATCGGACGTTTATGGGGATGGGTATCC |  |
| *Coxiella burnetii* | *icd* | Co_bu_icd_F | AGGCCCGTCCGTTATTTTACG | 74 |
|  |  | Co_bu_icd_R | CGGAAAATCACCATATTCACCTT |  |
|  |  | Co_bu_icd_P | TTCAGGCGTTTTGACCGGGCTTGGC |  |
| *Aplicomplexa* | *18S rRNA* | Apic_18S_F | TGAACGAGGAATGCCTAGTATG | 104 |
|  |  | Apic_18S_R | CACCGGATCACTCGATCGG |  |
|  |  | Apic_18S_P | TAGGAGCGACGGGCGGTGTGTAC |  |
| *Babesia microti* | *CCTeta* | Ba_mi_CCT_F | ACAATGGATTTTCCCCAGCAAAA | 145 |
|  |  | Ba_mi_CCT_R | GCGACATTTCGGCAACTTATATA |  |
|  |  | Ba_mi_CCT_P | TACTCTGGTGCAATGAGCGTATGGGTA |  |
| *Babesia canis (3 subspecies)* | *RNA 18S* | Ba_ca_18S_F | TGGCCGTTCTTAGTTGGTGG | 104 |
|  |  | Ba_ca_18S_R | AGAAGCAACCGGAAACTCAAATA |  |
|  |  | Ba_ca_18S_P | ACCGGCACTAGTTAGCAGGTTAAGGTC |  |
| *Babesia ovis* | *RNA 18S* | Ba_ov_18S_F | TCTGTGATGCCCTTAGATGTC | 92 |
|  |  | Ba_ov_18S_R | GCTGGTTACCCGCGCCTT |  |
|  |  | Ba_ov_18S_P | TCGGAGCGGGGTCAACTCGATGCAT |  |
| *Babesia divergens* | *hsp70* | Bab_di_hsp70_F | CTCATTGGTGACGCCGCTA | 83 |
|  |  | Bab_di_hsp70_R | CTCCTCCCGATAAGCCTCTT |  |
|  |  | Bab_di_hsp70_P | AGAACCAGGAGGCCCGTAACCCAGA |  |
| *Babesia sp. EU1* | *18S rRNA* | Ba_EU_18S_F | GCGCGCTACACTGATGCATT | 91 |
|  |  | Ba_EU_18S_R | CAAAAATCAATCCCCGTCACG |  |
|  |  | Ba_EU_18S_P | CATCGAGTTTAATCCTGTCCCGAAAGG |  |
| *Hepatozoon* spp. | *18S rRNA* | He_spp_18S_F | ATTGGCTTACCGTGGCAGTG | 175 |
|  |  | He_spp_18S_R | AAAGCATTTTAACTGCCTTGTATTG |  |
|  |  | He_spp_18S_P | ACGGTTAACGGGGGATTAGGGTTCGAT |  |
| *Hepatozoon canis* | *18S rRNA* | He_can_18S_F | TTCTAACAGTTTGAGAGAGGTAG | 221 |
|  |  | He_can_18S_R | AGCAGACCGGTTACTTTTAGC |  |
|  |  | He_can_18S_P | AGAACTTCAACTACGAGCTTTTTAACTGCAAC |  |
| *Hepatozoon americanum* | *18S rRNA* | He_ame_18S_F2 | GGTATCATTTTGGTGTGTTTTTAAC | 159 |
|  |  | He_ame_18S_R2 | CTTATTATTCCATGCTCCAGTATTC |  |
|  |  | He_ame_18S_P2 | AAAAGCGTAAAAGCCTGCTAAAAACACTCTAC |  |
| *Cytauxzoon felis* | *ITS2* | Cy_fel_ITS2_F | AAGATCCGAACGGAGTGAGG | 119 |
|  |  | Cy_fel_ITS2_R | GTAGTCTCACCCAATTTCAGG |  |
|  |  | Cy_fel_ITS2_P | AAGTGTGGGATGTACCGACGTGTGAG |  |
| *Rangelia vitalii* | *18S rRNA* | Ra_vit_18S_F | TAACCGTGCTAATTGTAGGGC | 92 |
|  |  | Ra_vit_18S_R | GAATCACCAAACCAAATGGAGG |  |
|  |  | Ra_vit_18S_S | TAATACACGTTCGAGGGCGCGTTTTGC |  |
| *Leishmania* spp. | *hps70* | Leish_spp_hsp70_F | CGACCTGTTCCGCAGCAC | 78 |
|  |  | Leish_spp_hsp70_R | TCGTGCACGGAGCGCTTG |  |
|  |  | Leish_spp_hsp70_P | TCCATCTTCGCGTCCTGCAGCACG |  |
| *Leishmania infantum* | *ITS* | Le_inf_ITS_F | CGCACCGCCTATACAAAAGC | 103 |
|  |  | Le_inf_ITS_R | GTTATGTGAGCCGTTATCCAC |  |
|  |  | Le_inf_ITS_P | ACACGCACCCACCCCGCCAAAAAC |  |
| *Theileria* spp. | *18S rRNA* | Th_spp_18S_F | TGAACGAGGAATGCCTAGTATG | 104 |
|  |  | Th_spp_18S_R | CACCGGATCACTCGATCGG |  |
|  |  | Th_spp_18S_P | TAGGAGCGACGGGCGGTGTGTAC |  |
| *Tick species* | *16S rRNA* | Tick_spp_16S_F | AAATACTCTAGGGATAACAGCGT | 99 |
|  |  | Tick_spp_16S_R | TCTTCATCAAACAAGTATCCTAATC |  |
|  |  | Tick_spp_16S_P | CAACATCGAGGTCGCAAACCATTTTGTCTA |  |
| *Rhipicephalus sanguineus* s.l. | *ITS2* | Rh_sa_ITS_F | TTGAACGCTACGGCAAAGCG | 110 |
|  |  | Rh_sa_ITS_R | CCATCACCTCGGTGCAGTC |  |
|  |  | Rh_sa_ITS_P | ACAAGGGCCGCTCGAAAGGCGAGA |  |
| *Escherichia coli* | *eae* | eae-F | CATTGATCAGGATTTTTCTGGTGATA | 102 |
|  |  | eae-R | CTCATGCGGAAATAGCCGTTA |  |
|  |  | eae-P | ATAGTCTCGCCAGTATTCGCCACCAATACC |  |
