# Supplementary material for: Microfluidic PCR and network analysis reveals complex tick-borne pathogen interactions in the tropics
Source: Parasit Vectors. 2024 Jan 4;17:5. doi: 10.1186/s13071-023-06098-0 (PMC10765916; doi:10.1186/s13071-023-06098-0)
Supplement: Supplementary file 4 — Additional file 4: Table 2. Primer pairs and PCR conditions used for validation of microfluidic real-time PCR results. [file 13071_2023_6098_MOESM4_ESM.docx]

**Additional file 1: Table 2.** Primer pairs and PCR conditions used for validation of microfluidic real-time PCR results.

| Pathogens | Primers sequences (5´- 3´) | Target gene | Amplicon size | PCR conditions | References |
| --- | --- | --- | --- | --- | --- |
| *Hepatozoon canis* | Outer primers |  |  | 35 cycles: |  |
|  | ATACATGAGCAAAATCTCAAC | 18S rRNA | 660 bp | 10 sec 98°C; 30 sec 50°C; 30 sec 72°C | Inokuma et al. (2002) |
|  | CTTATTATTCCATGCTGCAG |  |  |  |  |
|  | Inner primers |  |  | 35 cycles: |  |
|  | GGTATGGTATTGGCTTACC |  | 309 bp | 10 sec 98°C; 30 sec 51°C; 30 sec 72°C | Gomes et al. (2016) |
|  | CGAGCTTTTTAACTGCAACA |  |  |  |  |
| *Anaplasma* spp. / *Ehrlichia* spp. | Outer primers |  |  | 35 cycles: |  |
|  | GAACGAACGCTGGCGGCAAGC | 16S rRNA | 693 bp | 10 sec 98°C; 30 sec 60°C; 30 sec 72°C | Rar et al. (2005) |
|  | AGTA**Y**CG**R**ACCAGATAGCCGC |  |  |  |  |
|  | Inner primers |  |  | 35 cycles: |  |
|  | TGCATAGGAATCTACCTAGTAG |  | 629 bp | 10 sec 98°C; 30 sec 55°C; 30 sec 72°C |  |
|  | AGTA**Y**CG**R**ACCAGATAGCCGC |  |  |  |  |
| *Rickettsia* spp. | Outer primers |  |  | 35 cycles: |  |
|  | GTCAGCGTTACTTCTTCGATGC | *omp*B | 475 bp | 10 sec 98°C; 30 sec 57°C; 30 sec 72°C | Choi et al. (2005) |
|  | CCGTACTCCATCTTAGCATCAG |  |  |  |  |
|  | Inner primers |  |  | 35 cycles: |  |
|  | CCAATGGCAGGACTTAGCTACT |  | 267 bp | 10 sec 98°C; 30 sec 58°C; 30 sec 72°C |  |
|  | AGGCTGGCTGATACACGGAGTAA |  |  |  |  |
| *Babesia* spp. |  |  |  | 35 cycles: |  |
|  | GYY TTG TAA TTG GAA TGA TGG | 18S rRNA | 559 bp | 10 sec 98°C; 30 sec 61°C; 30 sec 72°C | Bonnet et al. (2007) |
|  | CCA AAG ACT TTG ATT TCT CTC |  |  |  |  |
| *Mycoplasma* spp. |  |  |  | 40 cycles: |  |
|  | TGCACCATCTGTCACTCTGTTAACCTC | 16S rRNA | 280 bp | 10 sec 98°C; 30 sec 55°C; 45 sec 72°C | van Kuppeveld et al. (1994) |
|  | GGGAGCAAACAGGATTAGATACCCT |  |  |  |  |

***** all PCR reactions: 3 min 98°C initial activation; 10 min 72°C final extension; **Y: T/C; R: A/G.**

-Choi, Y.J., Lee, S.H., Park, K.H., Koh, Y.S., Lee, K.H., Baik, H.S., Choi, M.S., Kim, I.S., Jang, W.J., 2005. Evaluation of PCR-based assay for diagnosis of spotted fever group rickettsiosis in human serum samples. Clinical and diagnostic laboratory immunology 12, 759-763.

-Gomes, L.d.A., Moraes, P.H.G., do Nascimento, L.d.C.S., O’Dwyer, L.H., Nunes, M.R.T., Rossi, A.d.R.P., Aguiar, D.C.F., Gonçalves, E.C., 2016. Molecular analysis reveals the diversity of Hepatozoon species naturally infecting domestic dogs in a northern region of Brazil. Ticks and tick-borne diseases 7, 1061-1066.

-Inokuma, H., Okuda, M., Ohno, K., Shimoda, K., Onishi, T., 2002. Analysis of the 18S rRNA gene sequence of a Hepatozoon detected in two Japanese dogs. Veterinary parasitology 106, 265-271.

-Rar, V.A., Fomenko, N.V., Dobrotvorsky, A.K., Livanova, N.N., Rudakova, S.A., Fedorov, E.G., Astanin, V.B., Morozova, O.V., 2005. Tick-borne pathogen detection, Western Siberia, Russia. Emerging infectious diseases 11, 1708-1715.

-van Kuppeveld, F.J.M.; Johansson, K.E.; Galama, J.M.D; Kissing, J.; Bölske, G.; van der Logt, J.T.M.; Melchers, W.J.G. Detection of mycoplasma contamination in cell cultures by a mycoplasma group-specific PCR. Applied Environment Microbiol. 1994, 60, 149–152.

-Bonnet, S.; Jouglin, M.; L’Hostis, M.; Chauvin, A. Babesia sp. EU1 from roe deer and transmission within Ixodes ricinus. Emerging infectious diseases. 2007, 13, 8, 1208.
